# Improvement of Phenolic Compounds, Essential Oil Content and Antioxidant Properties of Sweet Basil (*Ocimum basilicum* L.) Depending on Type and Concentration of Selenium Application

**DOI:** 10.3390/plants8110458

**Published:** 2019-10-29

**Authors:** Liubov Skrypnik, Anastasia Novikova, Elina Tokupova

**Affiliations:** Institute of Living Systems, Immanuel Kant Baltic Federal University, Universitetskaya str., 2, Kaliningrad 236040, Russia; AENovikova@stud.kantiana.ru (A.N.); ISHtrants@kantiana.ru (E.T.)

**Keywords:** selenium, basil, biofortification, essential oil, phenolic compounds, antioxidant activity

## Abstract

The effect of selenium biofortification on phytomass yield, selenium, essential oil and phenolic compounds content as well as antioxidant properties of basil leaves was investigated. Selenium in form of sodium selenate was applied either in nutrient solution or by foliar spraying at three levels (2.0, 5.0 and 10.0 μM). Selenium treatment significantly increased Se concentration in leaves up to 20.23 μg g^−1^ (addition in nutrient solution) and 10.74 μg g^−1^ (foliar application). Neither a toxic nor a beneficial effect of Se addition on the plants was observed. Se application of 2 µM Se in nutrient solution and of 5 µM Se by foliar spraying successfully enhanced production of essential oils, hydroxycinnamic acids, total phenolics and antioxidant activity of basil leaves. The anthocyanin content was positively affected only by application of Se in nutrient solution. Considering both an increase in the Se concentration in basil leaves and an increase in the production of phytonutrients, the optimal doses of selenium can be considered to be 5 and 10 μM for Se addition in nutrient solution and by foliar treatment, respectively. The results confirm the possibility of the enrichment of basil plants with selenium and thereby improving the nutritional qualities of the human diet.

## 1. Introduction

Basil (*Ocimum basilicum* L.) is an annual plant of the *Lamiaceae* family, growing wild in subtropical and tropical areas of America, Africa, Asia, and in some southern regions in Europe [1]. Today, basil belongs to worldwide cultivated aromatic plants. The cultivation of basil is performed under natural as well as greenhouse conditions. To increase the yield and to produce basil year round, cultivation in a greenhouse is more suitable than cultivation in an open field [2]. Furthermore, in comparison to traditional soil culture, hydroponic cultivation of basil has additional benefits, such as using less ground area to obtain a higher yield of biomass characterized by better quality properties [3].

Traditionally, basil has been used as a medicinal plant in the treatment of headaches, coughs, diarrhea, constipation, warts, worms, and kidney malfunction [4]. The medicinal properties of basil are associated with the presence in its leaves of a whole complex of biologically active compounds of various chemical structures [5]. In particular, it has been found that basil leaves are rich in phenolic acids (rosmarinic, chicoric, caffeic, and caftaric) [6], flavonol (quercetin, kaempferol) glycosides and anthocyanins [7,8]. The phenolic compounds listed above make the main contribution to the antioxidant properties of basil leaf extracts. Another important component of basil leaves and flowers is essential oil, which is of high value for the food and pharmaceutical application of this plant. The essential oils distilled from various basil cultivars can contain linalool, methyl chavicol, 1,8-cineole, eugenol, methyl eugenol, methyl isoeugenol, thymol, methyl cinnamate, citral, and camphor [9]. In several studies the antioxidant, antimicrobial, anti-inflammatory, antibacterial, antifungal activities as well as repellent, insecticidal, larvicidal and nematicidal activities of basil essential oils have been established [9,10,11].

The quantitative and qualitative compositions of the phytochemicals in basil leaves can vary within wide ranges and depend on both cultivation conditions and the variety of basil. Mineral plant nutrition is one of the main factors influencing plant metabolism and the level of secondary metabolites. Although selenium does not belong to essential trace elements for plants, at the same time there are a number of facts confirming the positive influence of selenium on various life processes of vegetative organisms [12,13]. In particular, it was shown that selenium biofortification increased the phenolic compound content, flavonoids and antioxidant activity in tomato fruits [14,15], broccoli [16], curly endive [17], garlic [18], and shallot [19]. Another aspect of plant biofortification with selenium is associated with its essentiality to human and animal nutrition. It has been shown that a low selenium dietary intake leads to cardiovascular disease, disorder of the endocrine system, and increased risk of cancer [17]. However, in terms of the selenium intake by humans as well as to ensure high plant productivity, it is very important to control the dosages of selenium fertilization. High doses of selenium can cause toxic effects, both in humans and in plant organisms. Basil does not belong to the group of plants that can accumulate high concentrations of selenium. On the one hand, this allows considering basil as a relative safe object for enrichment with selenium, but on the other hand, Se non-accumulator plants are more sensitive to increased doses of selenium, which can lead to a decrease in yield and plant death.

There are several studies on selenium enrichment of basil plants by different types of application. Several studies deal with the enhancement of the selenium content in basil plants through foliar fertilization [20,21,22,23] and addition of selenium to the nutrient solution [24,25,26]. In some of these studies, it was demonstrated that selenium fertilization not only increased the yield and selenium content in plants but also improved the phenolic compounds, essential oil content and antioxidant activity of basil leaf extracts [20,21,22,23].

To the best of our knowledge, the literature lacks information on effect of the interaction between selenium application type and dosage on quantitative and qualitative, especially antioxidative, properties of basil. In this regard, the present study was undertaken in an effort to investigate the accumulation of selenium, essential oils, phenolic compounds and the antioxidant activity in basil leaves by two types of selenium application (in nutrient solution and by foliar application) and three levels of selenium concentration (2.0, 5.0 and 10.0 μM).

## 2. Results

### 2.1. Plant Yield and Selenium Content

The untreated basil plants were characterized by a low selenium concentration in their leaves, accounting for 0.014 μg g^−1^ (Table 1). However, when using 2 μM selenium, the selenium concentration in the leaves increased by 75 times (by selenium application in a nutrient solution) and by 39 times (by foliar application). The use of higher doses (5 and 10 μM) of selenium contributed to a significant increase in the concentration in basil leaves. Furthermore, the method of selenium application also had a significant effect. By the selenium addition to the nutrient solution, its level in the leaves was 2–3 times higher than in the case of the foliar treatment at the same doses of selenium.

Despite such a significant increase in the selenium concentration in the treated plant leaves, no significant increase in yield and leaf biomass as compared to that of the control was observed (Table 1). No effect of selenium on the growth processes of basil plants was observed both by the selenium addition in the nutrient solution and by foliar application.

### 2.2. Essential Oil

**Figure 1:**
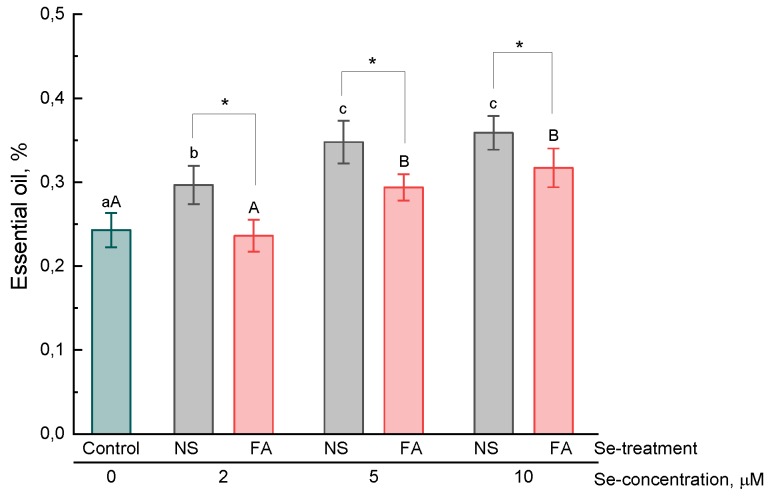
Effect of type and concentration of Se application on the essential oil content in basil leaves.

Selenium treatment of basil plants contributed to increase in the essential oil content in the leaves (Figure 1). By the addition of selenium into the nutrient solution (NS), a higher level of essential oil content as compared to that in control plants was observed even at 2 μM, whereas by foliar application (FA), a significant increase only occurred at 5 μM. At the same time, both by the addition of selenium into the nutrient solution and by the foliar application, no significant difference in the essential oil content in basil leaves grown at 5 and 10 μM was observed. The method of selenium application also had a significant effect on the accumulation of essential oil (Figure 1). A higher level was observed in plants that were grown in a nutrient solution containing selenium than in those that were treated with selenium by spraying.

### 2.3. Phenolic Compounds

In the present study, the effect of selenium addition on the accumulation of hydroxycinnamic acids, anthocyanins, flavonoids and total phenolics in basil leaves was studied. 

**Figure 2:**
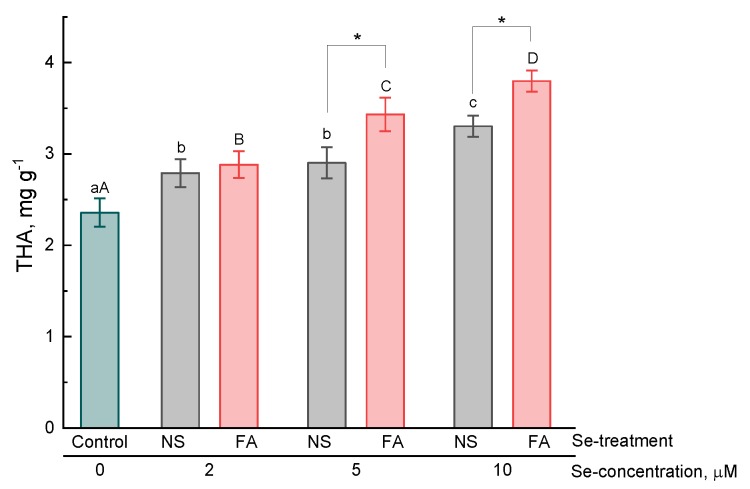
Effect of type and concentration of Se application on total hydroxycinnamic acid content (THA) in basil leaves.

By the treatment of basil plants with selenium, the level of hydroxycinnamic acids in the leaves was significantly higher as compared to that in untreated plants (Figure 2). The maximum content of hydroxycinnamic acids was observed in plants with foliar treatment of selenium at a concentration of 10 μM and exceeded that of the control values by 1.6 times. The accumulation of this class of compounds in basil leaves also depended on the type of treatment. For selenium dosages of 5 μM and 10 μM, significantly higher values were achieved in plants by foliar application as compared to that by the addition of selenium in the nutrient solution (Figure 2).

**Figure 3:**
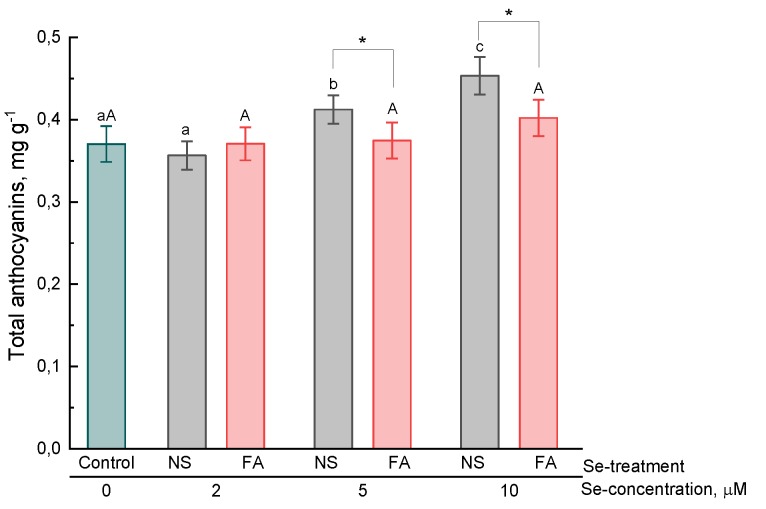
Effect of type and concentration of Se application on the total anthocyanin content in basil leaves.

The accumulation of high anthocyanin concentrations is more characteristic of purple basil; however, these pigments are also present in the leaf of green-leafed basil. The level of anthocyanins in the leaves of basil plants treated with selenium depended on the concentration of selenium and on the treatment type. Thus, a significantly higher content of these pigments as compared to that of control was observed in the leaves of basil grown in a nutrient solution containing 5 and 10 μM selenium (Figure 3). By foliar treatment, the level of anthocyanins in basil leaf did not significantly differ from that of control plants.

**Figure 4:**
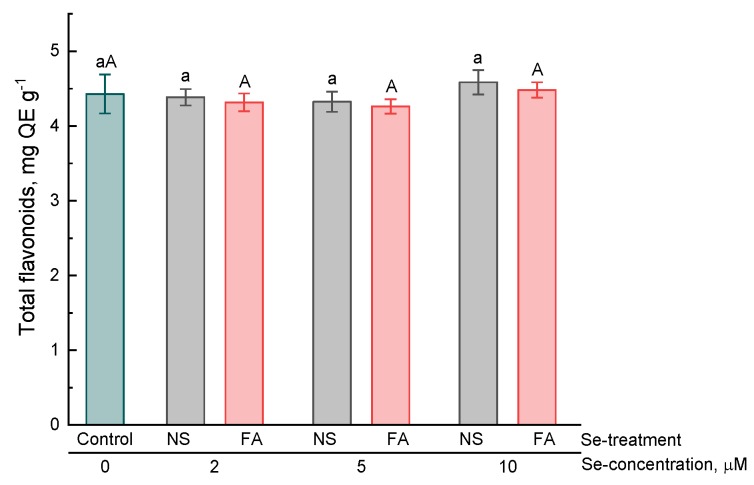
Effect of type and concentration of Se application on total flavonoid content in basil leaves.

Unlike the content of hydroxycinnamic acids and anthocyanins, the total level of flavonoids in basil leaves depended neither on the concentration of selenium nor on the type of selenium treatment and did not significantly differ from that in untreated plants (Figure 4).

**Figure 5:**
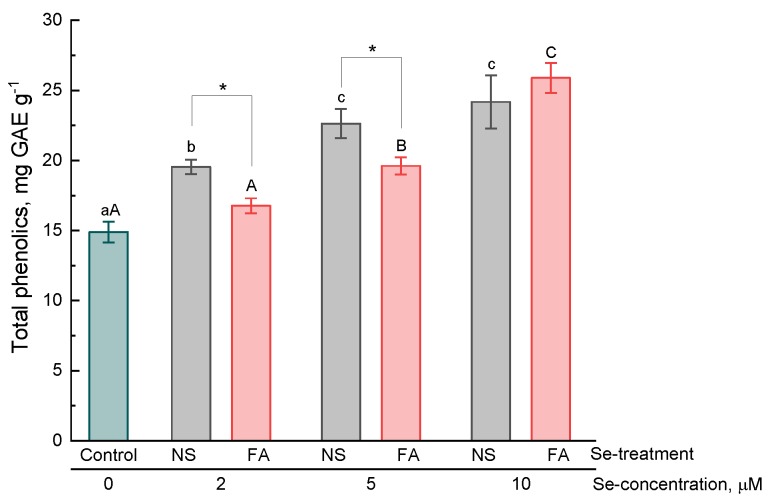
Effect of type and concentration of Se application on the total phenolic compound content in basil leaves.

Phenolic compounds are a class of secondary metabolites widely represented in plant organisms and include several thousand compounds with various structures (from simple phenolic acids to complex polymer compounds, and tannins). The study showed that the addition of 2 μM selenium to the nutrient solution contributed to an increase in the total phenolic compound content (TPC) as compared to that in control plants (Figure 5). However, foliar treatment at this concentration did not lead to significant changes in TPC in leaves. Differences from the control under this type of treatment were observed only at selenium concentrations of 5 μM and 10 μM. Thus, the type of treatment also affected the accumulation of total phenolic compounds. Higher values were observed by the addition of selenium to the nutrient solution at concentrations of 2 and 5 μM compared with foliar application at the same concentrations. When using a higher concentration of selenium (10 μM), there were no significant differences depending on the type of treatment, but plants of this particular variant were characterized by the maximum content of phenolic compounds in leaves.

### 2.4. Antioxidant Activity

Antioxidant activity is an integral indicator and depends on the presence of a whole complex of compounds in the extracts. The mechanism of the antioxidant action of these compounds may also be different. In this regard, in this study, three methods to evaluate the antioxidant activity of basil leaf extracts were employed. DPPH (1,1-diphenyl-2-picrylhydrazyl) and ABTS (2,2′-azino-bis(3-ethylbenzothiazoline-6-sulfonic acid) assays are based on the ability of antioxidants to bind radicals. The FRAP (ferric reducing antioxidant power) assay serves as an indicator of the reducing power of plant extracts. To compare the data obtained by all three methods, we used Trolox as a standard in this work. Results of studies of the effect of type and concentration of Se application on antioxidant activity of basil leaf extracts determined by the DPPH, ABTS and FRAP assays are presented in Table 2.

The obtained results show that selenium addition also affected the antioxidant activity of extracts from basil leaves. (Table 2). Despite the fact that the absolute values of antioxidant activity measured by the three methods were different, higher values were observed in plants treated with selenium. Furthermore, the antioxidant activity according to the DPPH method was significantly different from that of the control by the addition of 2 μM selenium into the nutrient solution. The antioxidant activity measured using ABTS and FRAP methods was significantly higher by selenium addition of 5 and 10 μM into the nutrient solution in comparison to the control. Foliar application of selenium led to a significant increase in the antioxidant activity of basil leaf extracts only when higher selenium concentrations were used (5 and 10 μM for DPPH and ABTS, 10 μM for FRAP). Two-way ANOVA showed that the type of treatment significantly influenced the leaf antioxidant activity measured by the DPPH and FRAP methods (Appendix A, Table A3). By the addition of selenium into the nutrient solution, the values of antioxidant activity were higher as compared to those by foliar treatment. For antioxidant activity according to the ABTS assay, no significant effect of the type of treatment was observed.

## 3. Discussion

Despite the fact that since 1957 selenium has been attributed as an essential microelement for humans, the experimental results of the studies of the effect of selenium supplements on the human body are contradictory [27,28]. Such contradictions are associated primarily with the dual nature of selenium, which is expressed in the fact that both a deficiency and an excess of this microelement are detrimental to human health [29]. The optimal plasma selenium status in most studies was around 120 ng L^−1^. A lower concentration of selenium in plasma increased the risk of developing some diseases, e.g., cancer, cardiovascular diseases, and diabetes [27]. The European Food Safety Authority recommends 55 μg day^−1^, as does the Food and Nutrition Board of the Institute of Medicine of the USA. The World Health Organization suggests 26 μg day^−1^ for females and 34 μg day^−1^ for males [28]. Selenium deficiency can be compensated by the introduction of additional amounts of selenium into the daily human diet. Plants are the first link in the human food chain; therefore, the development of technologies for the biofortification of plants with selenium can prevent a deficiency of this microelement in humans. However, since the concentration range between the beneficial and toxic effects of selenium is very narrow, a well-thought-out approach to choosing a plant species for enrichment with selenium, strict selection of selenium concentrations in fertilizers and studies on the accumulation of this microelement by a specific species or even plant variety are necessary. Basil does not belong to plants that can accumulate high concentrations of selenium. High concentrations of selenium are toxic to basil and induce the death of the plant. The literature provides conflicting data on the concentrations of selenium used for the biofortification of basil. In some studies the application of relatively high concentrations of selenium (from 10 to about 600 μM) for biofortification of basil was investigated [20,25,26]. However, Edelstein et al. (2016) demonstrated that Se concentrations in nutrient solution for basil plants should be lower than 0.25 mg L^−1^ (i.e., 3.2 μM) to avoid yield reduction and possible Se phytotoxicity [30]. In the present study, relatively low concentrations of selenium (2, 5, and 10 μM) were applied. The use of such low concentrations was supposed to ensure the safe accumulation of selenium both for plants and for humans. Thus, in this study, the maximum amount of selenium in basil leaves (20.23 μg g^−1^ DW or 1.61 μg g^−1^ FW) accumulated by the addition of selenium into the nutrient solution at a concentration of 10 μM was found. By foliar treatment at the same concentration, the content of selenium in basil leaves was approximately 2 times lower. Basil belongs to spice plants and is used as a spice in small quantities in dry or fresh form for flavoring vegetables, soups, meat, poultry and fish. The herb is famous for use in Italian dishes, such as pesto. The literature provides no data on the average amount of basil consumed daily. However, based on our data, the daily consumption of 2.7 g of dry or 34 g of fresh basil leaves enriched with 10 μM selenium could provide the amount of Se necessary to meet the recommended dietary allowances (RDA) (55 μg Se day^−1^). A consumption of about 200 g of Se-enriched fresh basil leaves would provide a higher amount of Se than the RDA, although this is considerably below the toxic threshold (400 g day^−1^).

In this study, neither a toxic nor positive effect of selenium addition on the biomass of basil plants was observed. The yield of selenium-treated plants did not significantly differ from that of the control variant. In selenium non-accumulator plants, the toxic effect of selenium is typically associated with the insertion of selenium into sulfur-containing amino acids and peptides instead of sulfur, which results in the disturbance of their function. In addition, selenium induces both oxidative and nitrosative stresses and thereby disturbs metabolism and damages cellular structures [31,32]. In the present study, there was no proteomic analysis that would allow us to evaluate the inclusion of selenium in the composition of organic molecules. However, the results indicate that treatment of basil plants with selenium both by adding selenium to the nutrient solution and by foliar application at a concentration not exceeding 10 μM did not result in a decrease in the yield of basil. A study of the effect of selenium on yield and the accumulation of selenium in basil plants cultivated in a nutrient solution containing selenium showed similar results [25]. In other studies [20,22], a significant change in the basil yield by foliar treatment with selenium was not shown. 

In the present study, it was found that the treatment of basil plants with selenium (introduction into a nutrient solution and foliar application) resulted in an increase in the content of essential oils, hydroxycinnamic acids, anthocyanins (only by the addition of selenium to solution), total content of phenolic compounds in the leaves as compared to untreated plants. Furthermore, regardless of the type of treatment and the concentration of selenium, the total content of flavonoids did not significantly differ from that of control plants. The results of our experiment partially correspond to those already available in the literature. For example, Lee et al. found that the essential oil content in selenium-treated (by introducing into a nutrient solution) basil plants was 2–3 times higher as compared to that of untreated ones [24]. In contrast, Mezeyova et al. [22] showed that foliar treatment of plants with selenium did not significantly increase the level of essential oil in basil leaves. There is evidence of an increase in the total phenolic compound content [21] and anthocyanins [33] in plant leaves by foliar treatment and the addition of selenium to nutrient medium, respectively. However, it should be noted that the mechanism of the effect of selenium on the secondary metabolism of plants remains poorly understood. An increase in the expression of the UFGT and F3H genes involved in the metabolism of anthocyanins, which results in an increase in the biosynthesis of these pigments, was shown in selenium-treated lettuce plants [33]. Zhu et al. [34] also showed that an increase in the level of flavonoids in tomatoes by the selenium treatment of plants was associated with an increase in the expression of a number of genes included in the biosynthesis of these secondary metabolites. However, the mechanisms of such regulation are not yet clear. On the one hand, an increase in the content of some phenolic compounds, for example, anthocyanins, in the leaves of selenium-treated plants may indicate the development of stress under the action of selenium and an increase in the biosynthesis of these components as the defense response in a plant cell. However, data on an increase in the content of some secondary metabolites were also obtained in experiments in which exogenous selenium was used in relatively low concentrations and no symptoms of toxicosis in plants were observed. In some cases, a decrease in malondialdehyde, which is a marker of lipid peroxidation, was recorded, which is more likely to indicate a decrease in oxidative processes in plant cells treated with low selenium concentrations [20,35]. These results are consistent with experimental data on the increase in antioxidant activity by the addition of selenium to a nutrient solution or by foliar application, as obtained both in present study and in early studies by some authors [16,25,36]. The increase in the antioxidant activity of plant extracts may be due, firstly, to an increase in the level of biosynthesis of phenolic or other secondary metabolites with antioxidant properties, secondly, to the effect of selenium on the redox metabolism of glutathione and enzymes involved in this metabolism, and thirdly, to the direct antioxidant effect of selenium itself and its organic metabolites.

## 4. Materials and Methods

### 4.1. Plant Materials and Growth Conditions

The experiment was conducted in a greenhouse at the Immanuel Kant Baltic Federal University (Kaliningrad, Russia), from 04 April 2016 to 10 June 2016 on sweet green-leaved basil (*Ocimum basilicum* L. cv Tonus). The basil seeds were sown on perlite and irrigated with half-strength nutrient solution. Fourteen-day-old seedlings of similar size were transferred to 2 L pots containing half-strength nutrition solution (four plants per pot). After a seven-day adaptation period, the plants were used for experiments. Two treatments, addition of selenium into the nutrient solution and foliar application, were investigated. Selenium was used in the form of a water solution of sodium selenate (Na_2_SeO_4_). The three levels of selenium (2.0, 5.0 and 10.0 μM) were studied. The treatments lasted 28 days. The renewing of the nutrient solution and foliar treatments with selenium were conducted every week. The control consisted of plants growing in nutrient solution without selenium and the concentrations of all other nutrients were as follows: 6.3 mM N-NO_3_, 0.7 mM N-NH_4_, 1.0 mM P-H_2_PO_4_, 1.0 mM S-SO_4_, 2.7 mM K, 2.5 mM Ca, 1.0 mM Mg, 50 μM Fe, 30 μM B, 4 μM Mn, 1 μM Cu, 1 μM Mo, 2 μM Zn. The nutrient solutions in each treatment had pH values of 5.7 and EC values of 2.3 dS m^−1^. pH and EC values were checked every 2 days and adjusted if necessary. 

Average air temperature in the greenhouse during the growing period was 18.2 °C, and the relative humidity was on average 72%. The mean daily (16 h) photosynthetically active radiation was 16.1 mol m^−2^ d^−1^ (light intensity 640 µmol m^−2^ s^−1^). The pots were arranged in a completely randomized design.

### 4.2. Plant Harvesting and Sample Preparation

Four weeks after initial selenium treatments, basil plants were harvested. The shoot and leaf biomasses were measured. One part of leaves from each plant was dried at 60 °C and used to determine the essential oil and selenium content. The other part of leaves was placed in liquid nitrogen within 5–10 min after harvesting and frozen at −80 °C. Later, it was lyophilized, ground and used for analysis of phenolic compounds and antioxidant activity.

### 4.3. Determination of Selenium in Basil

Total Se concentration was determined in oven-dried ground leaves by hydride generation atomic absorption spectrometry HG-AAS (SpectrAA 220 FS with hydride vapor generation accessory VGA 77, Varian) after mineralization of vegetative samples through autoclave decomposition under pressure [37]. Blank glass tubes contained all the chemical reagents without plant material or selenium standard solution. Total selenium concentration was expressed as μg per gram dry weight.

### 4.4. Isolation of the Essential Oil

The essential oil from oven-dried basil leaves was isolated by hydrodistillation for 4 h using a Clevenger-type apparatus [38]. The essential oil yield was calculated on a dry weight basis by a gravimetric method.

### 4.5. Determination of Total Anthocyanins, Hydroxycinnamic Acids, Flavonoids and Phenolic Compound Content

The total anthocyanin content in plant extracts was evaluated spectrophotometrically according to [39]. Plant extract preparation: 0.30 g lyophilized plant material was homogenized with 10 mL of extraction solvent (1% HCl water solution). The extracts were centrifuged for 30 min at 4500 rpm. The optical density of the above supernatant was determined at 510 nm (UV-3600, Shimadzu, Japan). To correct for the content of green pigments P.V. Maslennikov suggested to consider the optical density of the extracts obtained at 657 nm. As a standard, a solution of cyanidin-3-glucoside was used.

The total hydroxycinnamic acids, flavonoids and phenolic compound contents were determined in 96% ethanolic extracts prepared by homogenizing 0.1–0.2 g lyophilized plant material in 10 mL of 96% ethanol solution. The homogenates were centrifuged at 4500 g for 30 minutes. The supernatant was used for further analysis.

The total hydroxycinnamic acid (THA) content was evaluated as described in [40]. The reaction mixture contained 1.0 mL of the plant extract, 2 mL of 0.5 M HCl, 2 mL of chromogenic reagent, and 2 mL of 8.5% NaOH solution. The total volume of the reaction mixture was 10 mL, filled with dH_2_O. The chromogenic reagent was prepared by dissolving 10 g of NaNO_2_ and 10 g of Na_2_MoO_4_ in 100 ml of distilled water. The absorbance of the reaction mixture was measured at 505 nm (UV-3600, Shimadzu, Japan). The estimation of the total hydroxycinnamic acids content was performed using a calibration curve with rosmarinic acid solutions as standards. Results were expressed as mg per gram dry weight.

The determination of the total flavonoid content (TFC) was performed using a colorimetric assay according to Sevket et al. [41]. Into the 10 mL volumetric test tube, 4 mL of dH_2_O, 100 μL of the plant extract, 0.3 mL of 5% NaNO_2_ solution and 300 μL of 10% AlCl_3_ solution were added. After 6 minutes, 2 mL of 1 M NaOH solution was added and the total volume was filled up to 10 mL with dH_2_O. The absorbance of the reaction mixture was measured at 510 nm (UV-3600, Shimadzu, Japan). The estimation of the total flavonoid content was performed using a calibration curve and expressed as mg quercetin equivalent (QE) per gram dry weight.

The Folin-Ciocalteu method was used for the determination of the total phenolic compound content (TPC) [42]. A test tube containing 100 μL of plant extract and 300 μL of 0.2 M Folin-Ciocalteu reagent was incubated for 10 min at room temperature in darkness. After that, 6 mL of 6.75% Na_2_CO_3_ solution was added to the test tube. The absorbance of the reaction mixture was measured after 30 min of incubation (room temperature, darkness) at 765 nm (UV-3600, Shimadzu, Japan). The estimation of TPC was performed using a calibration curve with gallic acid solutions as standards. Results were expressed as mg gallic acid equivalent (GAE) per gram dry weight.

### 4.6. Determination of Antioxidant Activity Using the DPPH, ABTS and FRAP Assays

The total antioxidant activity of basil extracts was measured using DPPH (1,1-diphenyl-2-picrylhydrazyl) and ABTS^+^ (2,2′-azino-bis(3-ethylbenzothiazoline-6-sulfonic acid) radical scavenging capacity assays and ferric reducing antioxidant power (FRAP) assay as described previously by authors [43]. For analysis, 96% ethanolic extracts from lyophilized ground basil leaves were used. The antioxidant activity was expressed as mg Trolox equivalents (TE) per gram dry weight.

### 4.7. Statistical Analysis

The statistical processing of experimental data was carried out using SigmaPlot 12.3 (Systat Software GmbH, Erkrath, Germany). The mean of four independent samples was taken to represent the result of each replicate. Because the two-way ANOVA detected significant interactions between studied factors (Appendix A, Table A1, Table A2 and Table A3), one-way ANOVA was performed for each factor (Se-treatment and Se-concentration) separately. Before ANOVA procedures, data were checked for normality by the Shapiro–Wilk test and homogeneity of variance. The results were reported as mean ± standard deviation (*n* = 4). The graphs were created using OriginPro 9 (OriginLab Corporation, Northampton, MA, USA). In tables and on the graphs, different lower case letters indicate significant differences among plants due to selenium addition in nutrient solution; upper case letters indicate significant differences among plants due to selenium foliar application (*p* ≤ 0.05); asterisks * indicate significant differences among types of selenium application (*p* ≤ 0.05) based on post hoc Tukey’s tests.

## 5. Conclusions

Se biofortification of basil either in the form of supplementation to nutrient solution or by foliar spraying significantly influenced the selenium concentration in plant leaves and leaf nutraceutical compounds. A 5 μM addition of selenium to a nutrient solution and 10 μM for foliar selenium application are suggested as optimal concentrations. At these concentrations, the optimal combination of accumulation of selenium and biologically active components (essential oil, hydroxycinnamic acids, anthocyanins, sum of phenolic compounds, antioxidant activity) in basil leaves was observed. The lack of a significant effect of selenium on the accumulation of flavonoids requires further studies related to a more thorough study of the metabolism of selenium and to its role in regulating the biosynthesis of secondary metabolites at the molecular level. The results confirm the possibility of the enrichment of basil plants with selenium and the increase of Se concentration in the human diet, which is especially important for regions characterized by a low selenium content in soils, and, as a consequence, in food.

## Figures and Tables

**Table 1:** Effect of type and concentration of Se application on selenium concentration in basil leaves and yield of whole plants and leaves.

Se-Treatment	Se-Concentration, μM	Se Concentration in Leaves, μg g^−1^	Yield, g Plant^−1^	Leaves, g Plant^−1^
Control	0	0.014 ± 0.002 ^aA1^	2.71 ± 0.21 ^aA^	1.80 ± 0.12 ^aA^
Nutrient solution	2	1.06 ± 0.06 ^b,^*	2.70 ± 0.29 ^a^	1.82 ± 0.08 ^a^
	5	8.50 ± 0.350 ^c,^*	3.11 ± 0.22 ^a^	1.99 ± 0.19 ^a^
	10	20.23 ± 1.24 ^d,^*	2.80 ± 0.39 ^a^	1.95 ± 0.13 ^a^
Foliar application	2	0.55 ± 0.04 ^B,^*	2.72 ± 0.30 ^A^	1.82 ± 0.14 ^A^
	5	2.72 ± 0.11 ^C,^*	2.85 ± 0.29 ^A^	1.85 ± 0.13 ^A^
	10	10.74 ± 0.67 ^D,^*	3.12 ± 0.31 ^A^	2.03 ± 0.18 ^A^

^1^ Data were evaluated via one-way ANOVA for each factor (Se-treatment and Se-concentration) separately. Different lower case letters indicate significant differences among plants due to selenium addition in nutrient solution; upper case letters indicate significant differences among plants due to selenium foliar application (*p* ≤ 0.05); asterisks * indicate significant differences among types of selenium application (*p* ≤ 0.05) based on post hoc Tukey’s tests.

**Table 2:** Effect of type and concentration of Se application on antioxidant activity of basil leaf extracts determined by the DPPH, ABTS and FRAP assays.

Se-Treatment	Se-Concentration, μM	Antioxidant Activity, mg TE g^−1^		
		DPPH	ABTS	FRAP
**Control**	0	8.93 ± 0.26 ^aA1^	5.28 ± 0.13 ^aA^	15.51 ± 0.62 ^aA^
Nutrient solution	2	9.65 ± 0.23 ^b,^*	4.97 ± 0.27 ^a,^*	15.72 ± 0.48 ^a^
	5	11.59 ± 0.44 ^c,^*	6.60 ± 0.24 ^b,^*	20.75 ± 0.93 ^b,^*
	10	14.92 ± 0.56 ^d,^*	8.38 ± 0.18 ^c,^*	22.64 ± 1.22 ^c,^*
Foliar application	2	9.14 ± 0.27 ^A,^*	5.49 ± 0.25 ^A,^*	15.57 ± 0.36 ^A^
	5	10.27 ± 0.25 ^B,^*	6.32 ± 0.13 ^B,^*	16.76 ± 0.50 ^A,^*
	10	13.18 ± 0.22 ^C,^*	7.63 ± 0.25 ^C,^*	19.74 ± 0.61 ^B,^*

^1^ Data were evaluated via one-way ANOVA for each factor (Se-treatment and Se-concentration) separately. Different lower case letters indicate significant differences among plants due to selenium addition in nutrient solution; upper case letters indicate significant differences among plants due to selenium foliar application (*p* ≤ 0.05); asterisks * indicate significant differences among types of selenium application (*p* ≤ 0.05) based on post hoc Tukey’s tests.

## Appendix A

**Table A1:** Results of 2-factorial ANOVA for Se concentration in leaves and yield.

Main Effects	Factors	Se Concentration, µg g^−1^	Yield, g Plant^−1^	Leaves, g Plant^−1^
Se-treatment (T)	Nutrient solution	7.451 ^a^	2.829 ^a^	1.889 ^a^
	Foliar application	3.505 ^b^	2.851 ^a^	1.874 ^a^
Se-concentration	0	0.014 ^d^	2.706 ^a^	1.795 ^b^
(C)	2	0.805 ^c^	2.714 ^a^	1.816 ^ab^
	5	5.61 ^b^	2.983 ^a^	1.926 ^ab^
	10	15.48 ^a^	2.958 ^a^	1.990 ^a^
Significance	T	*	ns	ns
	C	*	ns	*
	T*C	*	ns	ns

Data were evaluated via two-way ANOVA, factors: type of selenium treatment and Se concentration, followed by Tukey’s HSD test (n = 4, *p* ≤ 0.05). Identical letters indicate that values do not differ significantly. Asterisks indicate significantly influential factors.

**Table A2:** Results of 2-factorial ANOVA for essential oils and the phenolic compound content in basil leaves under Se application.

Main Effects	Factors	EO, %	THA, mg g^−1^	TAC, mg g^−1^	TTC, mg g^−1^	TPC, mg g^−1^
Se-treatment (T)	Nutrient solution	0.312 ^a^	2.838 ^b^	0.398 ^a^	4.431 ^a^	20.31 ^a^
	Foliar application	0.273 ^b^	3.117 ^a^	0.379 ^b^	4.373 ^a^	19.29 ^b^
Se-concentration	0	0.243 ^b^	2.357 ^d^	0.370 ^bc^	4.428 ^ab^	14.89 ^d^
(C)	2	0.266 ^b^	2.836 ^c^	0.364 ^c^	4.351 ^ab^	18.15 ^c^
	5	0.321 ^a^	3.168 ^b^	0.393 ^b^	4.294 ^b^	21.12 ^b^
	10	0.338 ^a^	3.550 ^a^	0.428 ^a^	4.534 ^a^	25.03 ^a^
Significance	T	*	*	*	ns	*
	C	*	*	*	*	*
	T*C	*	*	*	ns	*

Data were evaluated via two-way ANOVA, factors: type of selenium treatment and Se concentration, followed by Tukey’s HSD test (n = 4, *p* ≤ 0.05). Identical letters indicate that values do not differ significantly. Asterisks indicate significantly influential factors. EO—essential oil, THA—total hydroxycinnamic acid content, TAC—total anthocyanin content, TFC—total flavonoid content, TPC—total phenolic content

**Table A3:** Results of 2-factorial ANOVA for antioxidant activity.

Main Effects	Factors	Antioxidant Activity, mg TE g^−1^		
		DPPH	ABTS	FRAP
Se-treatment (T)	Nutrient solution	11.27 ^a^	6.31 ^a^	18.65 ^a^
	Foliar application	10.38 ^b^	6.18 ^a^	16.90 ^b^
Se-concentration	0	8.93 ^d^	5.28 ^c^	15.51 ^c^
(C)	2	9.40 ^c^	5.23 ^c^	15.64 ^c^
	5	10.93 ^b^	6.46 ^b^	18.76 ^b^
	10	14.05 ^a^	8.00 ^a^	21.19 ^a^
Significance	T	*	ns	*
	C	*	*	*
	T*C	*	*	*

Data were evaluated via two-way ANOVA, factors: type of selenium treatment and Se concentration, followed by Tukey’s HSD test (n = 4, *p* ≤ 0.05). Identical letters indicate that values do not differ significantly. Asterisks indicate significantly influential factors.
